# An Epidemiological Model of Rift Valley Fever with Spatial Dynamics

**DOI:** 10.1155/2012/138757

**Published:** 2012-08-13

**Authors:** Tianchan Niu, Holly D. Gaff, Yiannis E. Papelis, David M. Hartley

**Affiliations:** ^1^Division of Integrated Biodefense, ISIS Center, Georgetown University Medical Center, Washington, DC 20007, USA; ^2^Fogarty International Center, National Institutes of Health, Bethesda, MD 20892-2220, USA; ^3^Department of Biological Sciences, Old Dominion University, Norfolk, VA 23529, USA; ^4^Virginia Modeling Analysis & Simulation Center, Old Dominion University, Suffolk, VA 23435, USA; ^5^Department of Microbiology and Immunology, Georgetown University Medical Center, Washington, DC 20007, USA

## Abstract

As a category A agent in the Center for Disease Control bioterrorism list, Rift Valley fever (RVF) is considered a major threat to the United States (USA). Should the pathogen be intentionally or unintentionally introduced to the continental USA, there is tremendous potential for economic damages due to loss of livestock, trade restrictions, and subsequent food supply chain disruptions. We have incorporated the effects of space into a mathematical model of RVF in order to study the dynamics of the pathogen spread as affected by the movement of humans, livestock, and mosquitoes. The model accounts for the horizontal transmission of Rift Valley fever virus (RVFV) between two mosquito and one livestock species, and mother-to-offspring transmission of virus in one of the mosquito species. Space effects are introduced by dividing geographic space into smaller patches and considering the patch-to-patch movement of species. For each patch, a system of ordinary differential equations models fractions of populations susceptible to, incubating, infectious with, or immune to RVFV. The main contribution of this work is a methodology for analyzing the likelihood of pathogen establishment should an introduction occur into an area devoid of RVF. Examples are provided for general and specific cases to illustrate the methodology.

## 1. Introduction

Rift Valley fever virus (RVFV; family: *Bunyaviridae*, genus *Phlebovirus*) is transmitted by mosquitoes and infects domestic livestock and humans in Africa and the Middle East [1]. The pathogen was first described in peer-reviewed research in [2] and was originally considered local to sub-Saharan and southern Africa regions [3]. However, the pathogen moved outside the sub-Saharan region with documented outbreaks in Egypt [4], Saudi Arabia, and Yemen [5]. Loss of human life during RVF outbreaks has been low, primarily affecting individuals in direct contact with animals. This changed in the 1973 epidemic that occurred in South Africa during which human deaths were documented [6]. Outbreaks in Egypt caused 958 human deaths in 1977 and 200 human deaths in 1987. The outbreaks in Kenya and Somalia in 1997 caused 478 human deaths [7]. In Yemen, estimates for human infections of the RVFV were estimated at over 1000 during August and November of 2000, with 121 recorded deaths. Unlike spread among humans that remains relatively low, RVFV spread among animals reaches epidemic proportions. In 1951 epizootics occurring in regions of high altitude in South Africa resulted in the death of an estimated 100,000 sheep [8]. The recent outbreak of RVF in South Africa caused a total of 92 human cases with 6 deaths, and over 50,000 animals are estimated to have been infected with over 1,500 reported to have died from RVF as at 6 April, 2010 [9]. In animals, infection can produce high rates of abortion and significant morbidity and mortality. Animal loss can lead to food shortages and economic impacts in outbreak periods.

Introduction of the pathogen to the continental US could have catastrophic results, in particular, because of the economic sensitivity of the US agriculture to any RVFV occurrence that would invariably lead to recalls, animal culling, and trade restrictions. The potential impact of RVF upon human and agricultural health highlights the importance of containment to endemic regions. Thus, it is important to develop analytic tools to understand the effect of transport to the dynamics of spatial spread and persistence of RVFV. In particular, quantitative methodologies for assessing how an importation of the virus into immunologically naive populations might propagate in both time and space are limited. Métras et al. have reviewed the modeling tools used to measure or model RVF risks in animals and discussed their contributions to increase the understanding of RVF occurrence or informed RVF surveillance and control strategies [10]. In order to obtain quantitative insights into the dynamics of RVFV, Mpeshe et al. formulated a deterministic model with mosquito, livestock, and human host as a system of nonlinear ordinary differential equations and provided numerical simulations to support the analytical results [11]. Gaff and her collaborators also applied compartmentalized multispecies deterministic model of RVF to study efficacy of countermeasures to disease transmission parameters [12].

In this work we describe the foundations of a mathematical approach to access spatial spread of an introduced RVFV. Our approach is based on a previous model of RVF transmission in a small local population, and multispecies epidemic models incorporating spatial structure more generally. A single *Aedes* mosquito is used to represent initial infection. We believe that a single infected *Aedes* mosquito is a reasonable approximation of initial introduction for studying the dynamics of RVFV spread. The RVF model of [3] considered *Aedes* mosquitoes, livestock (e.g., cattle, sheep, and goats), and *Culex* mosquitoes on a single patch, and identified the need to include spatial variation. This can be accomplished within the framework of [13] which models the epidemiological dynamics of arbitrary numbers of species occupying an arbitrary number of patches. Their approach includes patch-specific contact rates, incubation periods, and other biological factors. They also describe a method for computing the stability of the disease-free equilibrium in terms of the basic reproduction ratio. The work described here builds upon these two related efforts by constructing and analyzing a mathematical model of RVFV that includes both pathogen propagation within and spreading across different regions via the movement of humans, livestock, and mosquitoes. The model is analyzed to determine the stability and sensitivity of disease-free equilibrium and examples are provided to demonstrate the use of this approach in specific initial conditions.

## 2. Materials and Methods

The model described in [3] was constructed to describe the transmission of RVFV between three prototypes: two mosquito populations and one livestock population. The model considered both individual-to-individual transmission of virus between species (so-called “horizontal transmission”) and mother-to-offspring transmission of virus (vertical transmission) in one mosquito species. Let us term mosquitoes that can transmit RVFV both horizontally to livestock and vertically to their progeny “floodwater *Aedes*” mosquitoes and label this “species 1”. Livestock will be labeled “species 2”, and let us call mosquitoes that can transmit RVFV only horizontally to livestock “*Culex*” and label this “species 3”.

Consider populations of these species distributed throughout a large but finite two dimensional region. We can divide this large region into a lattice of distinct patches. Each patch may support subpopulations of each species. The general model allows for travel among any pair of patches in the simulated region. Travel between adjacent patches captures species moving across patch boundaries. Travel between disconnected patches captures situations in which species are transported across several patch boundaries without any likelihood of interaction with the environment except at the source and destination patch. An example of this movement is livestock that is transported from a farm to a different farm or auction house. Such travel need not be between adjacent patches; transportation may move individuals between one patch and a geographically disconnected patch. Species living on a given patch may have patch-specific epidemiologic and demographic characteristics.

**Figure 1:**
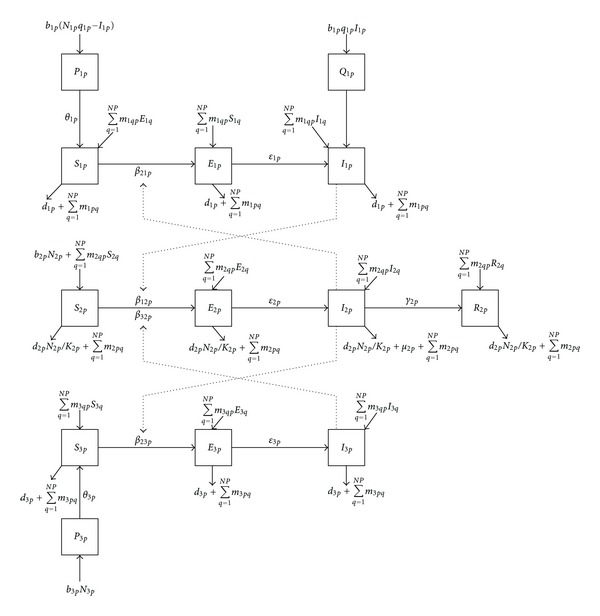
Flow diagram of the Rift Valley fever model with spatial dynamics.

We applied the methods of Arino et al. to the RVF model described in [3]. The resulting model is a system of ODEs describing the transmission of RVFV between the three generic species traveling between patches on a *P* × *P* rectangular lattice of patches.  Figure 1 is a schematic of the spatial and epidemiologic structure of our model.

Consider the *p*th patch in the lattice. On that patch infectious *Aedes* mosquitoes (species 1) transmit RVFV to susceptible livestock or they can become infected by taking a blood meal from infectious livestock hosts. They can also be infected vertically from infected mothers. *Culex* mosquitoes, species 3, can only transfer RVFV horizontally to livestock. Once infectious, mosquitoes remain so for the remainder of their lifespan. We assume that infection does not significantly affect mosquito behavior and longevity. Livestock (species 2) can become infected once bitten by infectious mosquitoes and they may die from RVFV infection or recover with life long immunity to RVFV infection. For each patch, the three populations have migrations from and to other patches, where they interact with similar populations in similar ways.

For patch *p*, the population of *Aedes* mosquitoes consists of uninfected (*P*_1*p*_) and infected (*Q*_1*p*_) eggs, and susceptible (*S*_1*p*_), incubating (infected, but not yet infectious) (*E*_1*p*_), and infectious (*I*_1*p*_) adult individuals. *Culex* mosquitoes consist of uninfected (*P*_3*p*_) eggs and susceptible (*S*_3*p*_), incubating (*E*_3*p*_) and infectious (*I*_3*p*_) adults. The size of each adult mosquito population is *N*_*ip*_ = *S*_*ip*_ + *E*_*ip*_ + *I*_*ip*_  (*i* = 1, 3). The livestock population consists of susceptible (*S*_2*p*_), incubating (*E*_2*p*_), infectious (*I*_2*p*_), and immune (*R*_2*p*_) individuals. The total livestock population size is *N*_2*p*_ = *S*_2*p*_ + *E*_2*p*_ + *I*_2*p*_ + *R*_2*p*_. The livestock population is logistic in nature with a carrying capacity *K*_2*p*_.

The resulting system of ODEs capturing the above epidemiological dynamics is shown below for *Aedes* mosquito vectors (see (1)–(6)), the livestock hosts (see (7)–(11)), and the *Culex* mosquito vectors (see (12)–(16)), respectively. The biological meaning of all model parameters is summarized in Table 1.

Consider the following:

(1) dP1pdt=b1p(N1p−q1pI1p)−θ1pP1p,

(2) dQ1pdt=b1pq1pI1p−θ1pQ1p,

(3) dS1pdtθ1pP1p−d1pS1p−β21pS1pI2pN2p +∑q=1Pm1qpS1q−∑q=1Pm1pqS1p,

(4) dE1pdt=β21pS1pI2pN2p−(d1p+ε1p)E1p +∑q=1Pm1qpE1q−∑q=1Pm1pqE1p,

(5) dI1pdt=θ1pQ1p−d1pI1p+ε1pE1p  +∑q=1Pm1qpI1q−∑q=1Pm1pqI1p,

(6) dN1pdtθ1p(P1p+Q1p)−d1pN1p  +∑q=1Pm1qpN1q−∑q=1Pm1pqN1p,

(7) dS2pdtb2pN2p−d2pS2pN2pK2p−β12pS2pI1pN1p−β32pS2pI3pN3p  +∑q=1Pm2qpS2q−∑q=1Pm2pqS2p,

(8) dE2pdt−d2pE2pN2pK2p+β12pS2pI1pN1p+β32pS2pI3pN3p−ε2pE2p  +∑q=1Pm2qpE2q−∑q=1Pm2pqE2p,

(9) dI2pdt=−d2pI2pN2pK2p+ε2pE2p−γ2pI2p−μ2pI2p +∑q=1Pm2qpI2q−∑q=1Pm2pqI2p,

(10) dR2pdt=−d2pR2pN2pK2p+γ2pI2p+∑q=1Pm2qpR2q−∑q=1Pm2pqR2p,

(11) dN2pdt=N2p(b2p−d2pN2pK2p)−μ2pI2p+∑q=1Pm2qpN2q−∑q=1Pm2pqN2p,

(12) $$\begin{array}{c} \frac{dP_{3p}}{dt}=b_{3p}N_{3p}-\theta_{3p}P_{3p}, \end{array}$$

(13) dS3pdt=θ3pP3p−d3pS3p−β23pS3pI2pN2p+∑q=1Pm3qpS3q−∑q=1Pm3pqS3p,

(14) dE3pdt=β23pS3pI2pN2p−(d3p+ε3p)E3p+∑q=1Pm3qpE3q−∑q=1Pm3pqE3p,

(15) dI3pdt=−d3pI3p+ε3pE3p+∑q=1Pm3qpI3q−∑q=1Pm3pqI3p,

(16) dN3pdt=(b3p−d3p)N3p+∑q=1Pm3qpN3q−∑q=1Pm3pqN3p.

### 2.1. Stability Analysis

There is an important quantity *ℛ*_0_ that expresses the stability of the disease free equilibrium (DFE) for epidemic models. The basic reproduction ratio *ℛ*_0_ is constructed in such a way as to express the number of secondary cases arising from a single primary infectious case in an entirely susceptible population [14, 15]. If *ℛ*_0_ < 1, it indicates that the infection cannot successfully transmit, on average, to one or more new host. The DFE is globally asymptotically stable as shown in Theorem 3.4 of reference [13]. If *ℛ*_0_ > 1, then the DFE is unstable and the pathogen may invade a susceptible population and persist [16]. The greater *ℛ*_0_ is above 1, the lower the likelihood of a newly-introduced pathogen fading out, although in the deterministic model presented in this study stochastic fadeout is not possible. Since the model of RVF has both vertical and horizontal transmission, *ℛ*_0_ for this system of ODEs is the sum of the *ℛ*_0_ values for each mode of transmission [17]: *ℛ*_0_ = *ℛ*_0,*V*_ + *ℛ*_0,*H*_. The first term *ℛ*_0,*V*_ represents the direct transmission, which is the vertical transfer of RVFV from infectious *Aedes* mosquito mothers to their offspring, whereas the second term *ℛ*_0,*H*_ is the indirect (vector borne) transmission, which is the transmission between vectors mediated by livestock hosts. By Theorem 3.4 in [13], we have the formula of *ℛ*_0,*H*_ for our model (formulas (17)–(25)) as *ℛ*_0,*H*_ = *ρ*(*T*_*H*_), where the next generation matrix *T*_*H*_ = *GB* ^−1^ *CA* ^−1^, *ρ*(*T*) represents the spectral radius of *T*, *ρ*(*T*) = max⁡_*j*_{|*λ*_*j*_|} and *λ*_*j*_ is the *j* eigenvalue of the matrix *T*. The matrices *G*, *B*, *C*, and *A* are defined as in [13].

The mobility matrix for the *𝒾* species is shown in (17). For the *i* species (*i* = 1,2, 3), the right null vector of the mobility matrix *M*_*i*_ under the constraint of total population *N*_*i*_^0^ is shown in (18). The matrix *G* is a diagonal block matrix defined in (19). The matrix *A* is a block matrix and each block is a diagonal matrix as shown in Equations (20) and (21). Matrix *B* is same as matrix *A* but with the incubation period replaced with the infection period. Matrix *C* is a diagonal block matrix defined in (22). Following [3] we calculate *ℛ*_0_ for vertical transmission by constructing the next generation matrix *T*_*V*_: *ℛ*_0,*V*_ = *ρ*(*T*_*V*_), where *T*_*V*_ = *FV* ^−1^. Matrix *F* is a diagonal block matrix and each block is a sparse matrix as shown in (24). Matrix *V* is also a diagonal block matrix, as shown in (25). Thus, *ℛ*_0_ = *ρ*(*GB* ^−1^ *CA* ^−1^) + *ρ*(*FV* ^−1^). This reduces to the expression for *ℛ*_0_ in Section  3 of [3] in the case of a single patch and no transportation. For arbitrary patch numbers *P* > 1, it must be evaluated numerically.

Consider,

(17) $$\begin{array}{c} M_{i}=\left[\begin{array}{cccc} -\sum_{q=1}^{P^{2}}m_{i1q} & m_{i21} & \cdots & m_{iP^{2}1}\\ \vdots & \vdots & \ddots & \vdots \\ m_{i1P^{2}} & m_{i2P^{2}} & \cdots & -\sum_{q=1}^{P^{2}}m_{iP^{2}q} \end{array}\right], \end{array}$$

(18) $$\begin{array}{c} N_{i}^{*}=\left(N_{i1}^{*},N_{i2}^{*},\ldots ,N_{iP^{2}}^{*}\right),\;N_{i}^{*}=\text{null}\left(M_{i}\right)N_{i}^{0}, \end{array}$$

(19) G=[G10⋯00G2⋯0⋮⋮⋱⋮00⋯GP2]= ⨁p=1P2Gp,Gp=(gijp)3×3, gijp=βijpNip∗Njp∗,  i,j=1,2,3,

(20) $$\begin{array}{c} A=\left[\begin{array}{cccc} A_{11} & A_{12} & \ldots & A_{1P^{2}}\\ A_{21} & A_{22} & \ldots & A_{2P^{2}}\\ \ldots & \ldots & \ldots & \ldots \\ A_{P^{2}1} & A_{P^{2}2} & \ldots & A_{P^{2}P^{2}} \end{array}\right]={\left(A_{jk}\right)}_{P^{2}\times P^{2}}, \end{array}$$

(21) Ajk=(aiijk)3×3,aiijk={dik+εik+∑l=1P2miklj=k,−mikjj≠k,

(22) C=[C10⋯00C2⋯0⋮⋮⋱⋮00⋯CP2]= ⨁p=1P2Cp,Cp=(ciip)3×3,  ciip=εip,

(23) $$\begin{array}{c} F=\left[\begin{array}{cccc} F_{1} & 0 & \cdots & 0\\ 0 & F_{2} & \cdots & 0\\ \vdots & \vdots & \ddots & \vdots \\ 0 & 0 & \cdots & F_{P^{2}} \end{array}\right]=\overset{P^{2}}{\underset{p=1}{⨁}}F_{p}, \end{array}$$

(24) $$\begin{array}{c} F_{p}={\left(f_{ij}^{p}\right)}_{3\times 3},\;\;f_{ij}^{p}=\{\begin{array}{cc} \theta_{1p} & i=3,j=1,\\ 0 & \text{others,} \end{array} \end{array}$$

(25) V=[V10⋯00V2⋯0⋮⋮⋱⋮00⋯VP2]= ⨁p=1P2Vp,Vp=[θ1p0−b1pq1p0d1p+ε1p00−ε1pd1p].

### 2.2. Model Sensitivity Analysis

Many of the parameters in this model, although they have biological interpretations, are either known imprecisely or vary significantly from region to region. Thus, it is of interest to investigate the sensitivity of the model to variation in parameter values. Here, we employed stochastic sampling from bounds placed on parameter estimates to assess the sensitivity of *ℛ*_0_ to model parameters. Specifically, we applied Latin hypercube sampling to get a large number of parameter sets based on the ranges of the parameters listed in Table 2. This approach has been used effectively in several other disease models [18–20]. Following [3], we assumed a uniform distribution for each parameter across its range in Table 2.

**Figure 2:**
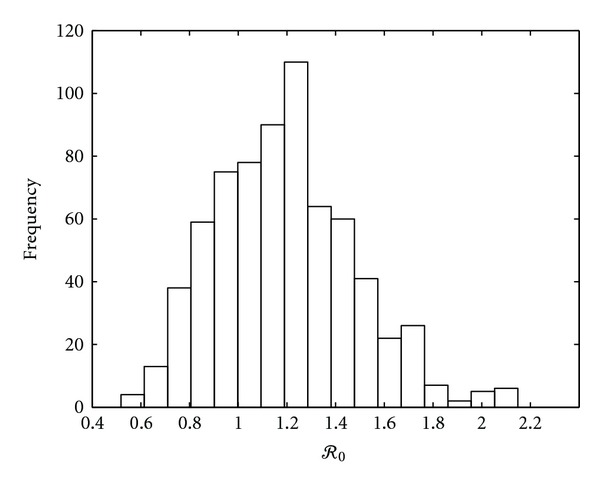
Distribution of *ℛ*_0_ values pooling a total of 700 sets of model parameters.

We calculated *ℛ*_0_ using *N* sets of sampled parameters for a 4-patch model as an example. Because our model includes *V* = 63 uncertain variables, *N* = 700 sets of sampled parameter values were generated by Latin hypercube sampling according to the suggestion of [21] that an *N* such that *N*/*V* > 10 should suffice for the number of stochastic samples of complete parameter sets. In this example, we used Latin hypercube sampling to generate the travel rates for 3 species (*m*_1*pq*_,  *m*_2*pq*_,  *m*_3*pq*_) and assumed that the travel rates from patch *p* to patch *q* are same as that from patch *q* to patch *p* (*m*_*ipq*_ = *m*_*iqp*_) for all *p* ≠ *q* and *i* = 1,2, 3.  Figure 2 is the histogram showing the resulting distribution of *ℛ*_0_. Averaging *ℛ*_0_ over all parameter sets gives a mean of 1.19 and a median of 1.18. *ℛ*_0_ ranged from 0.5 to 2.1.

We used the partial rank correlation coefficient (PRCC) to assess the significance of each parameter with respect to *ℛ*_0_. Partial rank correlation characterizes the linear relationship between rank-transformed inputs *X*_ranked_(*i*) and output *Y*_ranked_ after the linear effects on the output *Y*_ranked_ of the remaining inputs are discounted [22]. The results for the example above are shown in Table 3. The migration rates for all three populations appear to be significant with increasing migration decreasing *ℛ*_0_. This relationship is a result that the initial infection is only in one patch and could be diluted with increased mixing. Each of the four patches has same significant parameters, which are *β*_12*p*_, *β*_21*p*_, *β*_23*p*_, *β*_32*p*_, 1/*γ*_2*p*_, 1/*d*_1*p*_ and 1/*d*_3*p*_, (*p* = 1,…, 4). These PRCCs are all positive indicating an increase in *ℛ*_0_ with an increase in the adequate contact rate, mosquito lifespan, and the length of infection in livestock. The adequate contact rates of species 1 have a greater impact on *ℛ*_0_ than those of species 1 as a reflection of the biologically feasible range of each set of rates.

## 3. Numerical Simulation Results

Here, we consider a coupled lattice-based model by assuming a space consists of *P* × *P* patches, where each grid represents a subpopulation including *Aedes* mosquitoes, livestock, and *Culex* mosquitoes. In order to explore the transfer of RVFV on the space, we make the following simplifying assumptions. First, every patch has identical demographic and epidemiological parameter values. Second, only neighbored patches have nonzero travel rates, which are also balanced, *m*_*ipq*_ = *m*_*iqp*_ (*i* = 1, 2,3 and *p*, *q* = 1,…, *P* × *P*). The third assumption is that the initial population on each patch is of equal size [16].

### 3.1. Example 1: A 2 × 2 Space

We solved the model shown in (1)–(16) using a fourth-order Runge-Kutta scheme by choosing the time step to be 1 day. (We compared the RVFV prevalence in livestock on this space with different time step sizes (*h* = 1  day, 0.1 days, and 0.01 days), and found that our model is not sensitive to the step sizes.) The initial conditions are listed in Table 4. With the initial livestock population of 1000 animals, the patch size could be considered to be 4 square kilometers or 2 km by 2 km. Only the first patch has an infectious *Aedes* adult mosquito, all the other patches are free of RVFV at the beginning of time. In the horizontal transmission, the *β*_*ijp*_ have significant influence as shown in the significant test of parameters. Therefore, after reference [3], we used two sets of values for the adequate contact rates, *β*_*ijp*_, a relatively higher set and a lower set. There are also two types of livestock, one has higher RVF-associated mortality, like sheep, and one has lower RVF-associated mortality, like cattle. The time range was chosen to be 10 years, and we chose the lifespan of livestock to be 10 years, 5 years, and 2 years. The values of the parameters used in the four simulations are shown in Table 5.

**Figure 3:**
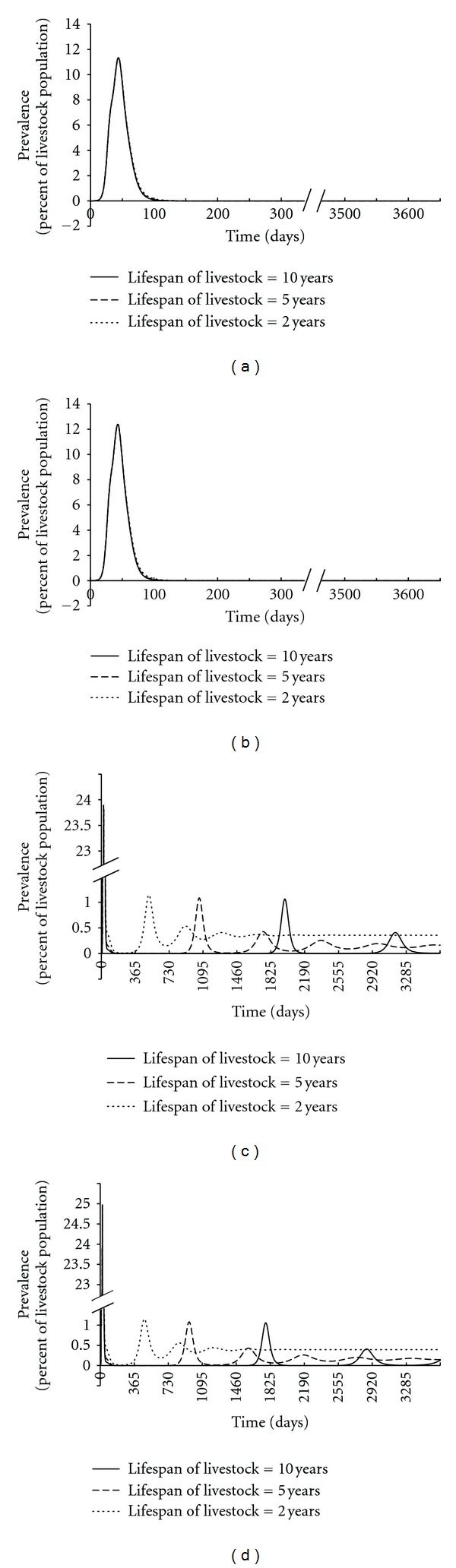
Results of numerical simulations for sheet and cattle with lower and higher set of contact rates. (a) Low contact rates and high RVFV related mortality rate. (b) Low contact rates and low RVFV-related mortality rate. (c) High contact rates and high RVFV related mortality rate. (d) High contact rates and low RVFV related mortality rate. Livestock lifespan is indicated for 10 years (solid line), 5 years (dashed line), and 2 years (dotted line).

Figure 3 depicts the percent of livestock infected through time on the whole space. For the simulations with relatively higher contact rates the initial outbreaks were large, so we need to break the  *y*-axis to show subsequent outbreaks clearly. For each case, the corresponding *ℛ*_0_ is shown in Table 6.  Figure 3(a) shows that with lower estimates of adequate contact rates *β*_*ijp*_ and the higher RVF-associated death rate (*μ*_2*p*_), after an initial epidemic reaching the maximum of 11.4%, RVFV is dying out for the rest of the time. We used lower estimates of contact rates and the lower RVF-associated death rate to generate Figure 3(b) and it is similar to that of Figure 3(a) with a slightly higher maximum prevalence of 12.4%.  Figure 3(c) shows that with higher *β*_*ijp*_ values and higher fatality estimates (*μ*_2*p*_), after an initial epidemic reaching 23.9% infected, there are subsequent epidemics with the final endemic level of between 0.1% and 1.5%.  Figure 3(d) shows that with higher *β*_*ijp*_ values and lower fatality estimates (*μ*_2*p*_) after an initial epidemic reaching 25.0% of the livestock infected, there are subsequent epidemics with the final endemic level between 0.1% and 1.5%. From Figures 3(c) and 3(d), it is obvious that with higher contact rates, more livestock could be infected and the RVF gains a foothold in the population. With shorter lifespan of livestock, less subsequent epidemics happen and the percent of infections of livestock turns to be stable faster. The expression for *ℛ*_0_ was confirmed numerically, that is, that for *ℛ*_0_ < 1, infections of RVFV dies out and for *ℛ*_0_ > 1, endemic states exist.

**Figure 4:**
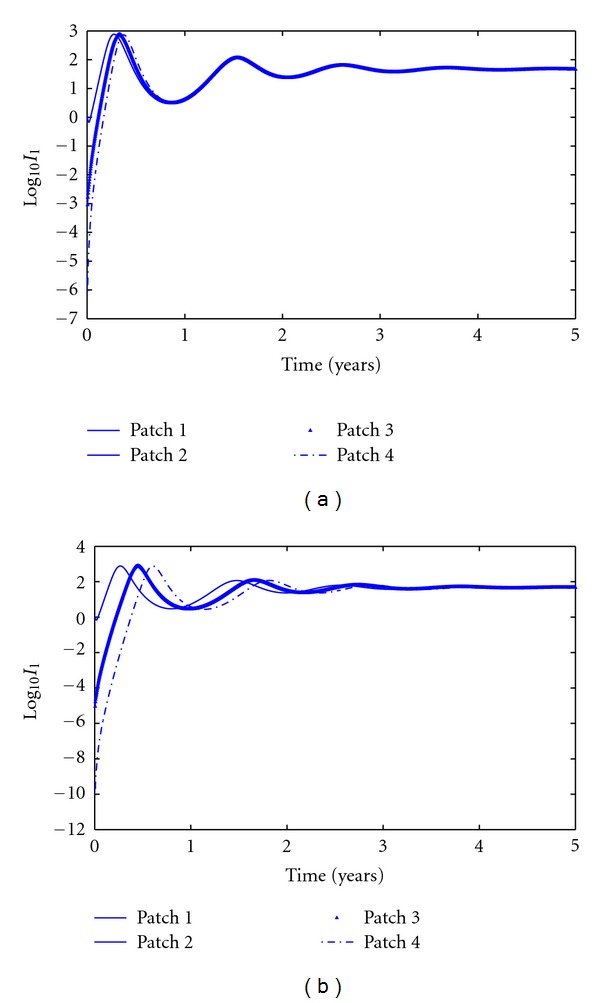
Simulations of a four-patch model with travel rates equal to (a) relatively larger travel rates: *m*_1_ = *m*_3_ = 0.001/day, *m*_2_ = 0.0025/day and (b) relatively smaller travel rates: *m*_1_ = *m*_3_ = 0.00001/day, *m*_2_ = 0.000025/day.

Figure 4 shows the simulations with (a) a larger set of travel rates (*m*_1_ = *m*_3_ = 0.001/day, *m*_2_ = 0.0025/day) and (b) a smaller set (*m*_1_ = *m*_3_ = 0.00001/day, *m*_2_ = 0.000025/day). The four curves show the numbers of infective *Aedes* mosquitoes on the four patches, plotted on a logarithmic scale. Both simulations have same initial conditions. At the beginning, there are antiphase oscillations and after a certain time, they are in phase. Comparing Figures 4(a) and 4(b), we can see that longer time taken for synchronization with the smaller travel rates. This is due to the high degree of mixing that occurs as time increases.

### 3.2. Example 1: A 20 × 20 Space

**Figure 5:**
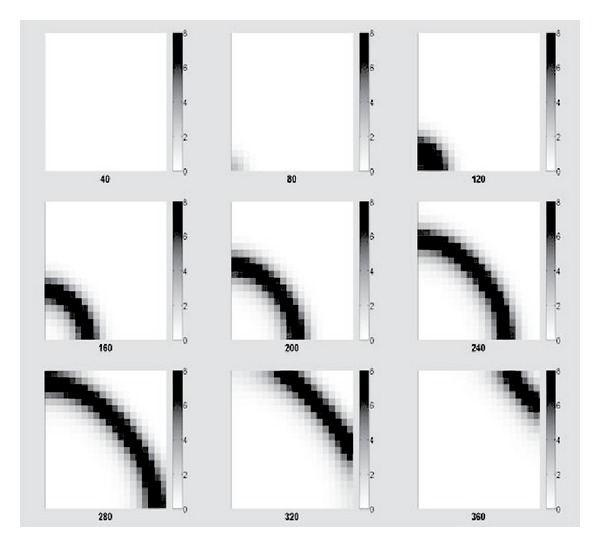
Snapshots of the space of 400 patches with initial infection on the 1st patch.

**Figure 6:**
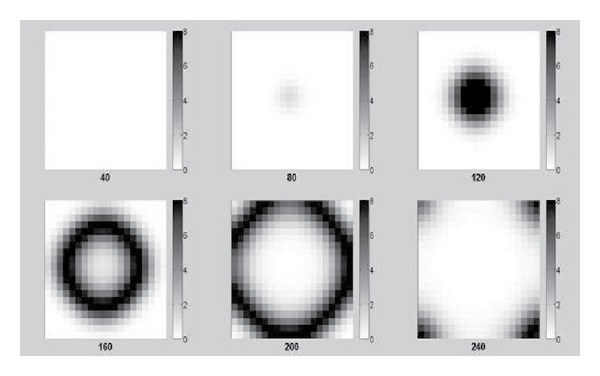
Snapshots of the space of 400 patches with initial infection on the center patch.

On a space of 400 patches, we solved the model similarly as for the 4-patch model except that we only focused on one simulation, which is with the higher set of *β*_*ijp*_, higher RVF-associated mortality and the 10-year lifespan of livestock. The first case is for the initial infection existing on the first patch, which is located at the left bottom corner of the space.  Figure 5 shows nine snapshots of the 400-grid space at different time points with darker shades illustrating a higher level of infection. It has a very clear fan-shaped wavelike spread of invading infections. The second case is the initial infection existing on the center patch of the space.  Figure 6 shows a circular wavelike spread of infection. Given the nearest neighbor only mobility, and the homogeneous populations on each patch, we expect the symmetric spread observed in Figures 5 and 6.

## 4. Spatial Heterogeneity

At different geographical locations, there are differences between populations of both vectors and hosts because of the environmental differences, which is referred as spatial heterogeneity [16]. For example, mosquito populations in different locations may experience differing habitat conditions that may affect their oviposition, blood meal choices, and other biological behaviors, which are connected with their birth rates, contact rates with hosts and so on; or different livestock populations may have different movement patterns leading to variation in spatial transmission rates. Spatial heterogeneity can affect the dynamics of the disease systems in many ways, therefore, in this section, we would like to consider the roles of the spatial heterogeneity playing in the persistence and transmission of the RVFV globally.

### 4.1. Effect of Space on Disease Persistence

The basic reproduction number *ℛ*_0_ is a key quantity in epidemiology, which essentially measures the average reproductive potential for an infectious disease [16]. Therefore, a threshold condition *ℛ*_0_ can tell us whether the pathogen will persist to the endemic state or die out in the long term.

For a space with *p* × *p* patches, we divide it into two regions: one with *ℛ*_0_ ≫ 1 and the other one with *ℛ*_0_ ≪ 1. In this case, HOT zones are placed on the lower left corner of the space. In order to explore the RVFV persistence on the whole space, *ℛ*_0_ was calculated for the whole space as the number of patches in the HOT zone increasing, which is represented by a radius concept with two variables *r*_*x*_ and *r*_*y*_ representing the dimension of the HOT zone rectangle on the lower left corner of the modeled environment. Here, we applied Latin hypercube sampling method to generate parameter values randomly for each patch in the HOT zone and the COOL zone based on the ranges of the parameter values in Table 2.

**Figure 7:**
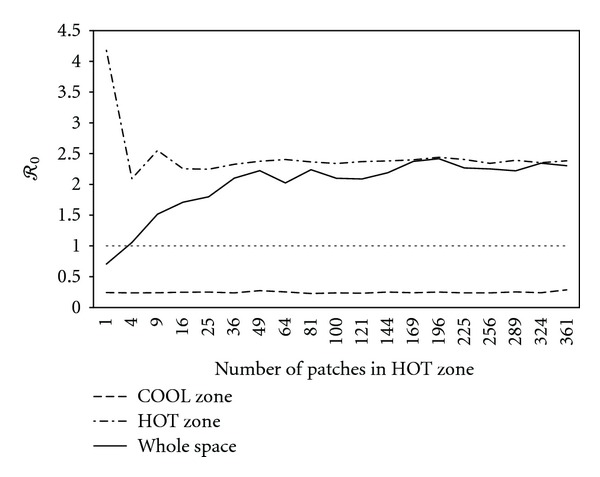
*ℛ*_0_ of the square HOT zone (dashed dot line), the COOL zone (dashed line) and the whole space (black line) on a 20 × 20-patch space as the number of patches in HOT zone increasing.

We first considered a simple case with the radius of the HOT zone being *r*_*x*_ = *r*_*y*_ = *r*, which is a square HOT zone. A space of 20 × 20 patches was taken as an example, and the results are shown in Figure 7. From the figure, it is clear that as the number of patches in HOT zone increasing, *ℛ*_0_ for the whole space is increasing from below 1 to meet *ℛ*_0_ of the HOT zone finally, since at last HOT zone occupies almost the whole space. The influences of the HOT zone on the whole space is dramatic.

**Figure 8:**
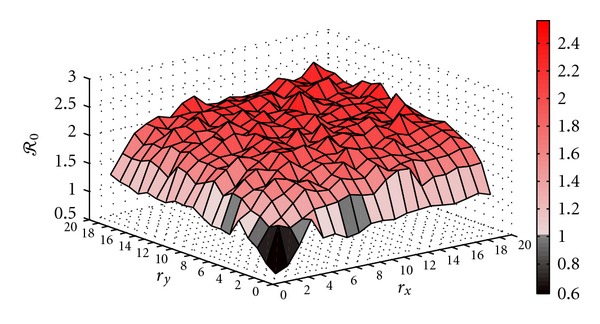
*ℛ*_0_ of the whole space on a 20 × 20-patch space as the number of patches in HOT zone increasing

Furthermore, a more general case with *r*_*x*_ ≠ *r*_*y*_ was simulated with the same 20 × 20 patch space. Results are depicted in Figure 8. *ℛ*_0_ of the whole space has similar pattern as shown in Figure 7, increasing as the HOT zone becoming larger.

**Figure 9:**
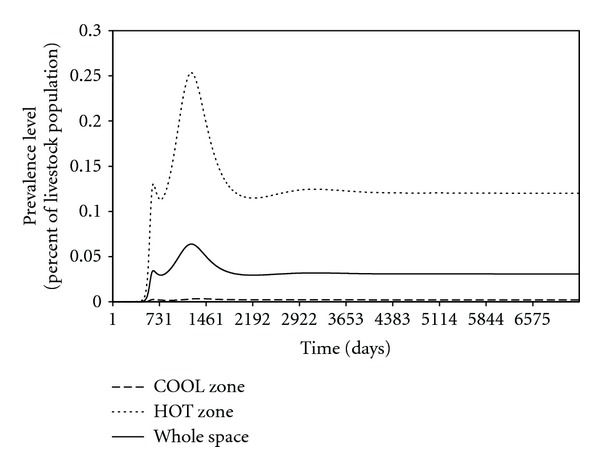
A prevalence figure of Rift Valley fever on a 20 × 20-patch space with a 100-patch HOT zone and a 300-patch COOL zone, whose parameter values are generated randomly by Latin hypercube sampling.

With the existence of spatial heterogeneity, even if the RVFV dies out locally, asynchrony between populations on different patches may allow global persistence [23]. For a 20 × 20 patch space *ℛ*_0_ = 2.0 with a 100-patch HOT zone (*ℛ*_0_ = 2.3) and a 300-patch COOL zone (*ℛ*_0_ = 0.5), whose parameter values are generated by Latin hypercube sampling, Figure 9 shows that the disease dies out on the COOL zone, but persists when considering the space as a whole.

### 4.2. Effect of Space on Disease Spread

In the previous sections, nearest neighbor movements of hosts and vectors between patches are considered. When long-distance traveling is not applicable, some natural barriers, like rivers or mountains, can play a very significant role on the spatial spread of infection. Therefore, in the following discussions, three rivers were introduced in the simulated space with 20 × 20 patches as arbitrary illustration of ecological barriers of directed migration. The initial condition consists of infection on the first patch located at the lower left corner of the modeled world. Two different scenarios about movements of hosts and vectors are considered. In the first scenario there are no rivers, hence, livestock and vectors travel is possible between adjacent patches. In the second scenario, three rivers located on the boundaries of patches prevent livestock and vectors from moving across these boundaries.

**Figure 10:**
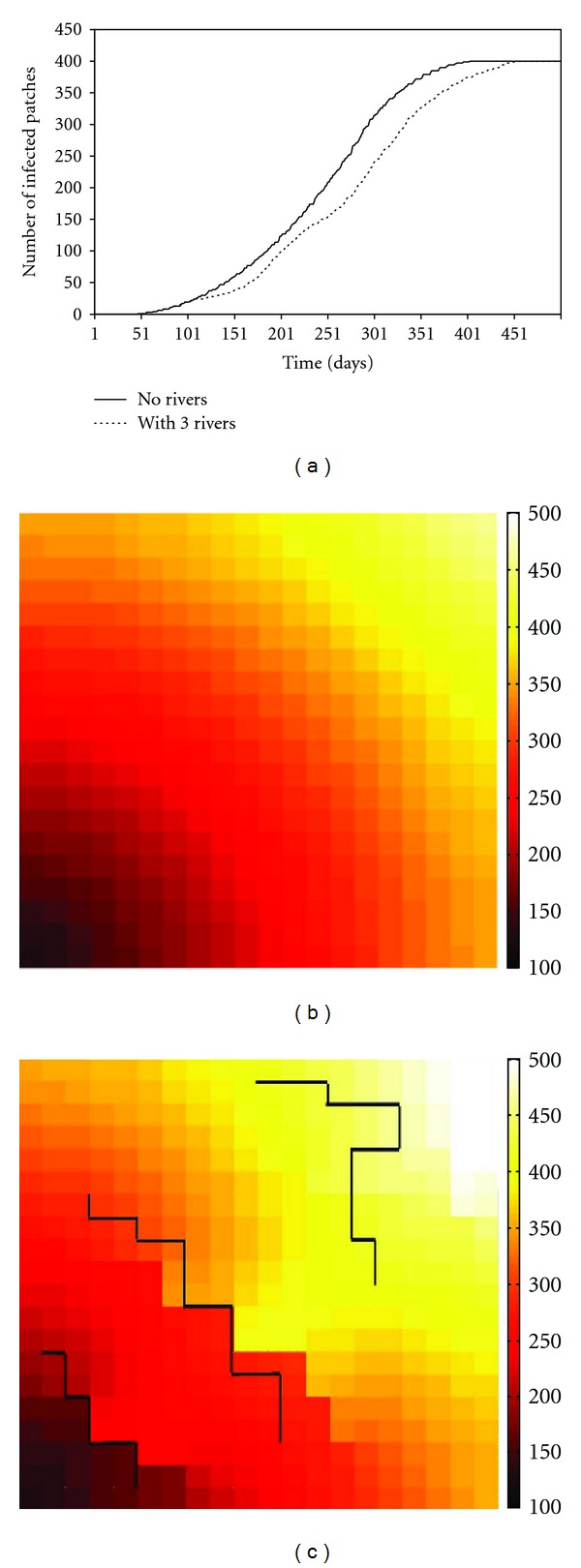
The effect of natural barriers (three rivers as an example here) on the spatial spread of Rift Valley fever on a 20 × 20 patch space.

Figure 10(a) depicts the number of infected patches as a function of time from the initial infection. When rivers exist, the spread speed of infection become lower and at the same time point, smaller number of patches got infected. By plotting the days of each patch taking to reach its outbreak peak, with darker colors representing earlier time in Figures 10(b) and 10(c), rivers clearly have a significant impact on the spatial spread of infection. When the impact of rivers is ignored (left map), the speed of spatial spread is far more rapid.

## 5. Conclusion

In this study, we built up an epidemiological model for Rift Valley fever on an arbitrary number of patches. Besides the transmission of RVFV between three prototypical species, our model considers the movements of hosts and vectors between patches, which cause the geographical transfer of the RVFV. Based on previous work, a formula to compute the basic reproduction ratio *ℛ*_0_ for the model was generated and used to analyze the sensitivity of the model and the stability of the disease free equilibrium. With example numerical simulations of the model, we illustrated the dynamics of the prevalence of RVFV on a space with grids and the spatial spread of RVF infections in some sample cases.

The model is a framework for simulating the spread of RVF or other similar disease transmitted by vectors and hosts on a multipatch geographic space connected by transportation. The simplifying assumptions adapted for the numerical simulations in this study would not necessarily be retained for simulations meant to model the real world. For example, in the case of RVF livestock may be transported via highway or rail to distant locations, which is inconsonant with the nearest-neighbor mobility assumption. Similarly, each patch may have patch-specific epidemiologic model parameters, reflecting population heterogeneities and environmental variation. In such cases, the symmetric disease waves observed in Figures 5 and 6 will become distorted.

As in reference [3], this model represents considerable simplification of the complex epidemiology of RVF. However, in this study we have removed one of simplifications in the prior work, namely, the assumption that spatial effects do not matter. Two outstanding issues remain to be addressed: inclusion of age structure and inclusion of public health control measures. Gaff et al. have constructed a single-patch mathematical model of RVF to access the efficacy of countermeasures on livestock population and emphasized the need to include spatial heterogeneous settings [12]. We hope this study will be of help to researchers who wish to address such needs.

## Figures and Tables

**Table 1:** Biological meaning of model parameters.

Parameter	Description	Unit
*β*_12*p*_	Adequate contact rate from *Aedes* to livestock on patch *p*	1/day
*β*_21*p*_	Adequate contact rate from livestock to *Aedes* on patch *p*	1/day
*β*_23*p*_	Adequate contact rate from livestock to *Culex* on patch *p*	1/day
*β*_32*p*_	Adequate contact rate from *Culex* to livestock on patch *p*	1/day
1/*d*_1*p*_	Lifespan of *Aedes* mosquitoes on patch *p*	Day
1/*d*_2*p*_	Lifespan of livestock on patch *p*	Day
1/*d*_3*p*_	Lifespan of *Culex* mosquitoes on patch *p*	Day
*b*_1*p*_	Number of *Aedes* eggs laid per day on patch *p*	1/day
*b*_2*p*_	Daily birthrate of livestock on patch *p*	1/day
*b*_3*p*_	Number of *Culex* eggs laid per day on patch *p*	1/day
*K*_2*p*_	Carrying capacity of livestock on patch *p*	Heads
1/*ε*_1*p*_	Incubation period in *Aedes* mosquitoes on patch *p*	Day
1/*ε*_2*p*_	Incubation period in livestock on patch *p*	Day
1/*ε*_3*p*_	Incubation period in *Culex* mosquitoes on patch *p*	Day
1/*γ*_2*p*_	Infectiousness period in livestock on patch *p*	Day
1/*μ*_2*p*_	RVF mortality rate in livestock on patch *p*	1/day
*q*_1*p*_	Transovarial transmission fraction in *Aedes* on patch *p*	Proportion
1/*θ*_1*p*_	Development time of *Aedes* on patch *p*	Day
1/*θ*_3*p*_	Development time of *Culex* on patch *p*	Day
*m*_1*pq*_	Travel rate of *Aedes* mosquitoes from patch *p* to patch *q*	1/day
*m*_2*pq*_	Travel rate of livestock from patch *p* to patch *q*	1/day
*m*_3*pq*_	Travel rate of *Culex* mosquitoes from patch *p* to patch *q*	1/day

**Table 2:** Parameters with estimated ranges for numerical simulations.

Parameter	(Range)	Reference
*β*_12*p*_	(0.0021, 0.2762)	[24–30]
*β*_21*p*_	(0.0021, 0.2429)	[24–28, 31, 32]
*β*_23*p*_	(0.0000, 0.3200)	[25–28, 31–33]
*β*_32*p*_	(0.0000, 0.0960)	[25–28, 33]
1/*d*_1*p*_	(3, 60)	[28, 34, 35]
1/*d*_2*p*_	(360, 3600)	[36]
1/*d*_3*p*_	(3, 60)	[28, 34, 35]
*b*_1*p*_	*d*_1*p*_	
*b*_2*p*_	*d*_2*p*_	
*b*_3*p*_	*d*_3*p*_	
1/*ε*_1*p*_	(4, 8)	[37]
1/*ε*_2*p*_	(1, 6)	[6]
1/*ε*_3*p*_	(4, 8)	[37]
1/*γ*_2*p*_	(1, 5)	[38]
*μ*_2*p*_	(0.025, 0.1)	[6, 38]
*q*_1*p*_	(0.0, 0.1)	[39]
1/*θ*_1*p*_	(5, 15)	[28]
1/*θ*_3*p*_	(5, 15)	[28]
*m*_1*pq*_	(0, 1)	[16]
*m*_2*pq*_	(0, 1)	[16]
*m*_2*pq*_	(0, 1)	[16]

**Table 3:** Significance test of parameters using PRCC.

Parameter	PRCC				*P* values
*m*_1*pq*_	−0.2283				<0.0001
*m*_2*pq*_	−0.1547				<0.0001
*m*_3*pq*_	−0.1376				0.0005

	*p* = 1	*p* = 2	*p* = 3	*p* = 4	

*β*_12*p*_	0.3965	0.3235	0.3125	0.3594	<0.0001
*β*_21*p*_	0.3259	0.3271	0.3752	0.3103	<0.0001
*β*_23*p*_	0.1972	0.1973	0.2058	0.2083	<0.0001
*β*_32*p*_	0.2149	0.2170	0.1706	0.1590	<0.0001
1/*γ*_2*p*_	0.3491	0.2777	0.3216	0.3276	<0.0001
1/*d*_1*p*_	0.4474	0.4320	0.3983	0.1590	<0.0001
1/*d*_3*p*_	0.2345	0.1679	0.1779	0.2809	<0.0001

**Table 4:** Initial conditions of the 4-patch RVF model.

	Patch 1	Patch 2, 3, and 4
*P*_1_(0)	5000	5000
*Q*_1_(0)	0	0
*S*_1_(0)	4999	5000
*E*_1_(0)	0	0
*I*_1_(0)	1	0
*N*_1_(0)	5000	5000
*S*_2_(0)	1000	1000
*E*_2_(0)	0	0
*I*_2_(0)	0	0
*R*_2_(0)	0	0
*N*_2_(0)	1000	1000
*P*_3_(0)	5000	5000
*S*_3_(0)	5000	5000
*E*_3_(0)	0	0
*I*_3_(0)	0	0
*N*_3_(0)	5000	5000

**Table 5:** Parameter values used in the simulations.

Parameter	Higher set	Lower set	
*β*_12*p*_	0.480	0.150	
*β*_21*p*_	0.395	0.150	
*β*_23*p*_	0.560	0.150	
*β*_32*p*_	0.130	0.050	
*μ*_2*p*_	0.0312	0.0176	
1/*d*_1*p*_			10
1/*d*_2*p*_			10 × 365, 5 × 365, 2 × 365
1/*d*_3*p*_			10
*b*_1*p*_			*d*_1*p*_
*b*_2*p*_			*d*_2*p*_
*b*_3*p*_			*d*_3*p*_
1/*ε*_1*p*_			6
1/*ε*_2*p*_			4
1/*ε*_3*p*_			6
1/*γ*_2*p*_			4
*q*_1*p*_			0.05
*θ*_1*p*_			0.1
*θ*_3*p*_			0.1
*K*_2*p*_			1000
*m*_1*pq*_			0.001
*m*_2*pq*_			0.0025
*m*_3*pq*_			0.001

**Table 6:** *ℛ*_0_ for the four simulations.

Simulation cases	*ℛ*_0_		
	1/*d*_2*p*_ = 10 years	1/*d*_2*p*_ = 5 years	1/*d*_2*p*_ = 2 years
Lower *β*_*ijp*_ & higher fatality rate	0.8005	0.7998	0.7976
Lower *β*_*ijp*_ & lower fatality rate	0.8405	0.8397	0.8372
Higher *β*_*ijp*_ & higher fatality rate	2.2930	2.2908	2.2842
Higher *β*_*ijp*_ & lower fatality rate	2.4112	2.4087	2.4015
